# Greater survival benefits in small cell lung cancer patients: a real-world study with immune checkpoint inhibitors therapy

**DOI:** 10.3389/fonc.2026.1940634

**Published:** 2026-09-24

**Authors:** Penghu Gao, Hongbing Zhang, Minghui Liu, Jinghao Liu, Zihe Zhang, Xuanguang Li, Yu Hua, Yongwen Li, Honglin Zhao, Hongyu Liu, Jun Chen

**Affiliations:** 1 Department of Lung Cancer Surgery, Center of Thoracic Surgery, Tianjin Medical University General Hospital, Tianjin, China; 2 Tianjin Key Laboratory of Lung Cancer Metastasis and Tumor Microenvironment, Tianjin Lung Cancer Institute, Tianjin Medical University General Hospital, Tianjin, China

**Keywords:** immune checkpoint inhibitor, immunotherapy, real-world study, small-cell lung cancer, survival analysis

## Abstract

**Background:**

Small-cell lung cancer (SCLC) is a highly aggressive neuroendocrine malignancy. Immune checkpoint inhibitors (ICIs) combined with chemotherapy have become a standard first-line treatment for extensive-stage SCLC; however, their effectiveness across different clinical settings in real-world practice remains incompletely characterized. This study aimed to retrospectively evaluate the clinical effectiveness of ICI-based therapy across subgroups defined by disease stage, surgery, and radiotherapy status.

**Methods:**

Patients with histologically or cytologically confirmed SCLC treated at Tianjin Medical University General Hospital between January 2010 and December 2024 were retrospectively included and classified into ICI and non-ICI groups according to treatment exposure. The primary endpoints were overall survival (OS) and progression-free survival (PFS), and the secondary endpoint was the 12-month objective response rate (ORR). Survival outcomes were estimated using the Kaplan–Meier method and compared using the log-rank test. Multivariable Cox proportional hazards regression was performed to adjust for clinically relevant covariates.

**Results:**

A total of 142 patients were included, including 76 in the ICI group and 66 in the non-ICI group. ICI therapy was associated with significantly longer OS in patients with limited-stage SCLC (52 vs. 24 months; HR = 0.31, P = 0.011), extensive-stage SCLC (28 vs. 12 months; HR = 0.29, P = 0.007), stage IIIB–C disease (46 vs. 21 months; HR = 0.37, P = 0.038), and stage IV disease (30 vs. 11 months; HR = 0.26, P = 0.007). Significant PFS improvements were observed primarily in patients with stage IV disease (9 vs. 5 months; HR = 0.31, P = 0.002) and extensive-stage disease (10 vs. 5 months; HR = 0.36, P = 0.003). A significantly higher ORR was observed in the ES-SCLC subgroup, while a numerical increase was observed in the stage IV subgroup. Subgroup analyses further suggested more pronounced survival improvements associated with ICI therapy among patients who did not undergo surgery or radiotherapy.

**Conclusions:**

ICI-based therapy was associated with improved survival outcomes in patients with SCLC, with the most consistent benefits observed in advanced-stage disease. More apparent treatment-associated benefits were also observed among patients who did not undergo surgery or radiotherapy. These findings provide real-world evidence supporting the use of ICI-based therapy in SCLC, particularly in advanced-stage disease, but should be interpreted cautiously given the retrospective design and subgroup sample sizes.

## Introduction

Small cell lung cancer (SCLC) is a highly aggressive neuroendocrine malignancy accounting for approximately 10%–15% of all lung cancers and is characterized by rapid progression, early metastasis, and poor prognosis (1, 2). Approximately 70% of patients are diagnosed with extensive-stage disease at presentation (3–5). For decades, platinum-etoposide–based chemotherapy has remained the standard first-line treatment for extensive-stage small cell lung cancer (ES-SCLC). However, despite high initial response rates, long-term survival remains poor, with a median overall survival of only approximately 10 months (6, 7).

In recent years, immune checkpoint inhibitors (ICIs) have significantly changed the therapeutic landscape of SCLC. Landmark phase III trials, including IMpower133, CASPIAN, and CAPSTONE-1, demonstrated that PD-1/PD-L1 inhibitors combined with platinum-etoposide chemotherapy significantly improve survival outcomes in ES-SCLC patients (7, 8). Based on these findings, ICI therapy combined with chemotherapy have been recommended as the first-line treatment for advanced-stage SCLC (9, 10). However, despite these advances, the prognosis of SCLC remains poor, and substantial heterogeneity in treatment response persists. Moreover, randomized clinical trials often apply strict enrollment criteria, limiting the generalizability of their findings to real-world populations (11, 12).

Therefore, in the present study, we retrospectively analyzed patients with SCLC treated at our institution to evaluate the real-world efficacy of ICI-based therapy across different disease stages and treatment backgrounds.

## Data and methods

### Patient selection and data acquisition

Patients diagnosed with SCLC at the Department of Lung Cancer Surgery, Tianjin Medical University General Hospital between January 2010 and December 2024 were retrospectively enrolled in this study. Eligible patients met the following criteria: (1) histologically or cytologically confirmed SCLC; (2) initiation of first-line treatment at our institution; (3) receipt of at least three cycles of systemic anti-tumor therapy; (4) availability of complete clinical data; and (5) age ≥18 years. Patients with mixed histology, concurrent active malignancies, incomplete medical records, or participation in other interventional clinical trials were excluded. The patient selection process is illustrated in **Figure 1**.

**Figure 1:**
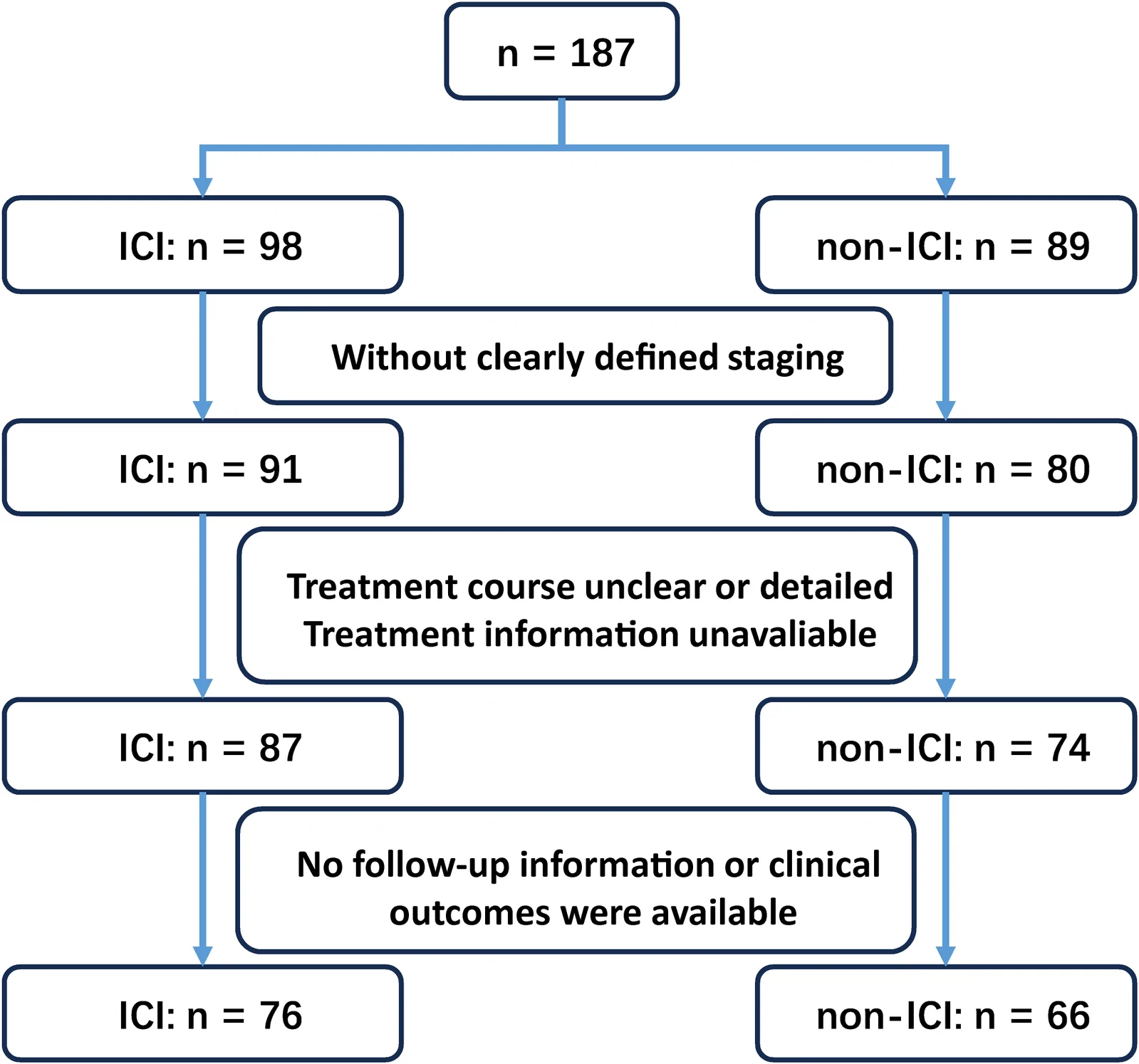
Flowchart of patient selection and study cohort construction. Patients were screened according to the predefined inclusion criteria, and eligible patients were subsequently included in the final analysis.

Patients were stratified into an ICI group (n = 76) and a non-ICI group (n = 66) according to treatment exposure. Patients who received at least three cycles of first-line PD-1/PD-L1 inhibitor combined with platinum–etoposide chemotherapy were assigned to the ICI group, whereas those who received platinum–etoposide chemotherapy without ICI were assigned to the non-ICI group. Clinical and pathological data were retrieved from the institutional electronic medical record system.

### Outcomes and assessments

Patients who initiated first-line systemic treatment between January 2010 and December 2024 were retrospectively included. December 2024 marked the end of enrollment, whereas clinical follow-up continued thereafter, with the database updated through April 2026.

The primary endpoints were progression-free survival (PFS) and overall survival (OS). PFS was defined as the time from initiation of first-line treatment to radiographic progression or death from any cause, whichever occurred first. OS was defined as the time from treatment initiation to death from any cause.

The secondary endpoint was the 12-month objective response rate (ORR), defined as the proportion of patients classified as having complete response (CR) or partial response (PR) at the 12-month response assessment. Tumor response was evaluated according to RECIST 1.1 using a predefined hierarchical approach. Patients with radiographically confirmed progressive disease (PD) before 12 months or tumor-related death before the 12-month assessment were classified as non-responders. For patients who remained alive and progression-free, response status was determined using CT performed within 12 months ± 2 weeks after treatment initiation; when multiple examinations were available, the scan closest to 12 months was selected. At the 12-month assessment, CR and PR were classified as responses, whereas stable disease (SD) and PD were classified as non-responses. No confirmatory CT examination was required.

### Statistical analysis

Statistical analyses were performed using R software (version 4.3.1), SPSS (version 28.0), and GraphPad Prism (version 10). Continuous variables were summarized as mean ± standard deviation or median (interquartile range), as appropriate, and categorical variables as counts and percentages. Between-group comparisons of categorical variables, including the 12-month ORR, were performed using Pearson’s chi-square test without continuity correction or Fisher’s exact test, as appropriate. Fisher’s exact test was used when any expected cell count was <5.

Survival outcomes were estimated using the Kaplan–Meier method and compared using the log-rank test. Hazard ratios (HRs) and 95% confidence intervals (CIs) were estimated using Cox proportional hazards regression. The proportional hazards assumption was assessed using Schoenfeld residuals, with no significant violations identified.

All statistical tests were two-sided, and P < 0.05 was considered statistically significant.

## Results

### Clinical characteristics

A total of 142 patients with SCLC were included in the study, with 66 patients in the non-ICI group and 76 in the ICI group. Clinical characteristics are summarized in **Table 1**. Tumor location and radiotherapy exposure were relatively well balanced between the two groups, whereas modest imbalances were observed in age, sex, and smoking status, with more pronounced imbalances in disease stage and surgical treatment. Stage IV disease was present in 42.1% of patients in the ICI group compared with 21.2% in the non-ICI group, while ES-SCLC accounted for 51.3% and 25.8%, respectively. Surgical resection was less common in the ICI group (10.5% vs. 51.5%). For a small number of patients, the laterality of the primary or biopsied lesion could not be reliably determined from the available clinical records, including 4 patients in the ICI group and 3 in the non-ICI group.

**Table 1:** Clinical characteristics of research cohort.

Category	ICI	Non-ICI	SMD
Age			0.189
>70	13 (17.1%)	7 (10.6%)	
≤70	63 (82.9%)	59 (89.4%)	
Sex			0.182
Male	58 (76.3%)	45 (68.2%)	
Female	18 (23.7%)	21 (31.8%)	
Smoking Status			0.136
Ever	51 (67.1%)	40 (60.6%)	
Never	25 (32.9%)	26 (39.4%)	
Tumor Location			0.046
Left	32 (42.1%)	29 (43.9%)	
Right	40 (52.6%)	34 (51.5%)	
Missing/Unknown	4 (5.3%)	3 (4.5%)	
Clinical Stage			
III	44 (57.9%)	52 (78.8%)	0.461
IV	32 (42.1%)	14 (21.2%)	
Limited Stage	37 (48.7%)	49 (74.2%)	0.544
Extensive Stage	39 (51.3%)	17 (25.8%)	
Radiation			0.030
No	54 (71.1%)	46 (69.7%)	
Yes	22 (28.9%)	20 (30.3%)	
Surgery			0.988
Yes	8 (10.5%)	34 (51.5%)	
No	68 (89.5%)	32 (48.5%)	

ICI, immune checkpoint inhibitor; SMD, standardized mean difference; VALSG, Veterans Administration Lung Study Group. SMDs were calculated to assess baseline balance between the ICI and non-ICI groups. An absolute SMD <0.10 was considered indicative of adequate balance. For tumor location, missing data were treated as a separate category when calculating the overall SMD.

Regarding treatment response, CR, PR, SD, and PD were observed in 9 (11.8%), 31 (40.8%), 1 (1.3%), and 35 (46.1%) patients in the ICI group, respectively, compared with 17 (25.8%), 11 (16.7%), 0, and 38 (57.5%) patients in the non-ICI group. Accordingly, 40 of 76 patients (52.6%) in the ICI group and 28 of 66 patients (42.4%) in the non-ICI group achieved an objective response (CR or PR).

The patient enrollment period ended in December 2024, whereas clinical follow-up continued thereafter. At the final data cutoff in April 2026, survival and treatment-response data were updated for the study cohort, 54 deaths and 88 disease progression events had occurred. Among patients with disease progression, post-treatment progression in the ICI group involved the thoracic/primary tumor site in 16 patients, brain in 4, abdomen in 5, multiple sites in 15, bone in 2, and cervical region in 1. In the non-ICI group, progression involved the primary tumor site in 25 patients, brain in 6, abdomen in 1, multiple sites in 12, and bone in 1. These sites represent patterns of disease progression after treatment rather than baseline metastatic sites.

### Subgroup analysis reveals consistent survival benefits of ICI in SCLC

ICI treatment was associated with OS benefits across different disease stages and treatment backgrounds in patients with SCLC. Stratified analysis by disease stage demonstrated that ICI therapy significantly prolonged mOS in patients with LS-SCLC (52 months for ICI vs. 24 months for non-ICI, HR = 0.31, 95% CI: 0.13–0.77, P = 0.011, **Figure 2A**), with 1-year OS rates of 100% and 73.2%, respectively. Similar survival benefits were observed in patients with ES-SCLC (28 months for ICI vs. 12 months for non-ICI, HR = 0.29, 95% CI: 0.12–0.71, P = 0.007, **Figure 2B**), with corresponding 1-year OS rates of 85.8% and 49.4%, respectively. Further subgroup analysis revealed significant OS improvement in patients with stage IIIB–C disease (46 months for ICI vs. 21 months for non-ICI, HR = 0.37, 95% CI: 0.15–0.95, P = 0.038, **Figure 2C**) and stage IV disease (30 months for ICI vs. 11 months for non-ICI, HR = 0.26, 95% CI: 0.10–0.69, P = 0.007, **Figure 2D**). Although patients with stage IIIA disease also exhibited a trend toward prolonged survival (62 months for ICI vs. 36 months for non-ICI), the difference did not reach statistical significance.

**Figure 2:**
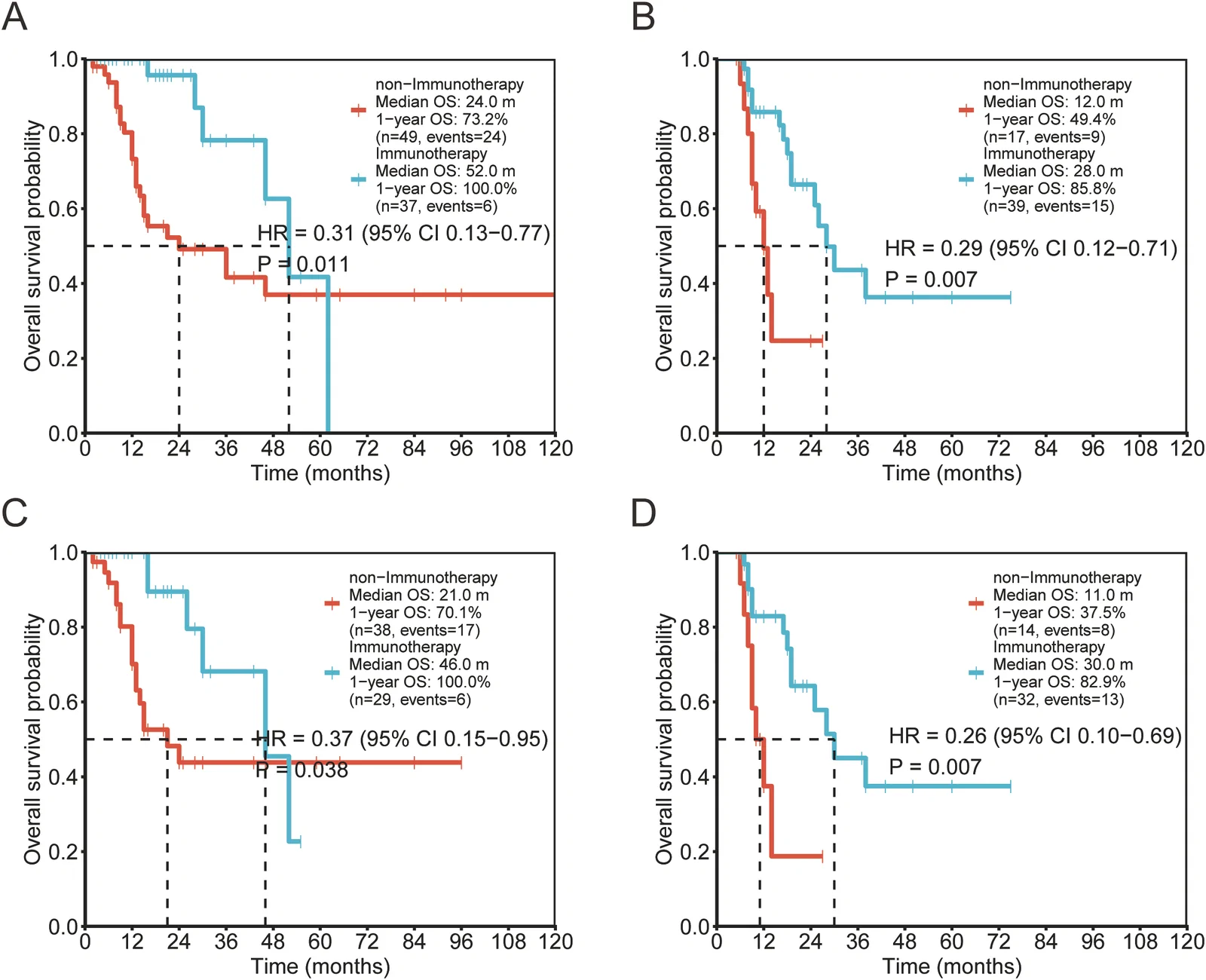
ICI therapy improved overall survival across different disease stages in patients with SCLC. Kaplan–Meier analyses of overall survival (OS) in patients treated with or without immune checkpoint inhibitor (ICI) therapy. Significant OS benefits with ICI treatment were observed in patients with LS-SCLC **(A)**, ES-SCLC **(B)**, stage IIIB–C disease **(C)**, and stage IV disease **(D)**. Patients with stage IIIA disease also showed a trend toward prolonged survival, although the difference was not statistically significant. HR, hazard ratio; OS, overall survival; ICI, immune checkpoint inhibitor.

Among patients who did not undergo surgical resection, ICI treatment significantly improved OS in both LS-SCLC (not reached (NR) vs. 21 months for non-ICI, HR = 0.15, 95% CI: 0.04–0.54, P = 0.004; 1-year OS: 100% vs. 71.1%, **Figure 3A**) and ES-SCLC patients (30 months for ICI vs. 14 months for non-ICI, HR = 0.29, 95% CI: 0.09–0.86, P = 0.026; 1-year OS: 88.3% vs. 53.3%, **Figure 3B**). Consistent survival benefits were also observed in stage III (46 months for ICI vs. 21 months for non-ICI, HR = 0.21, 95% CI: 0.07–0.62, P = 0.005; 1-year OS: 100% vs. 72.2%, **Figure 3C**) and stage IV disease (30 months for ICI vs. 12 months for non-ICI, HR = 0.28, 95% CI: 0.09–0.88, P = 0.030; 1-year OS: 85.8% vs. 50%, **Figure 3D**). In contrast, among patients who underwent surgical resection, ICI therapy showed a numerical trend toward prolonged OS in the LS and stage III subgroups, although statistical significance was not reached.

**Figure 3:**
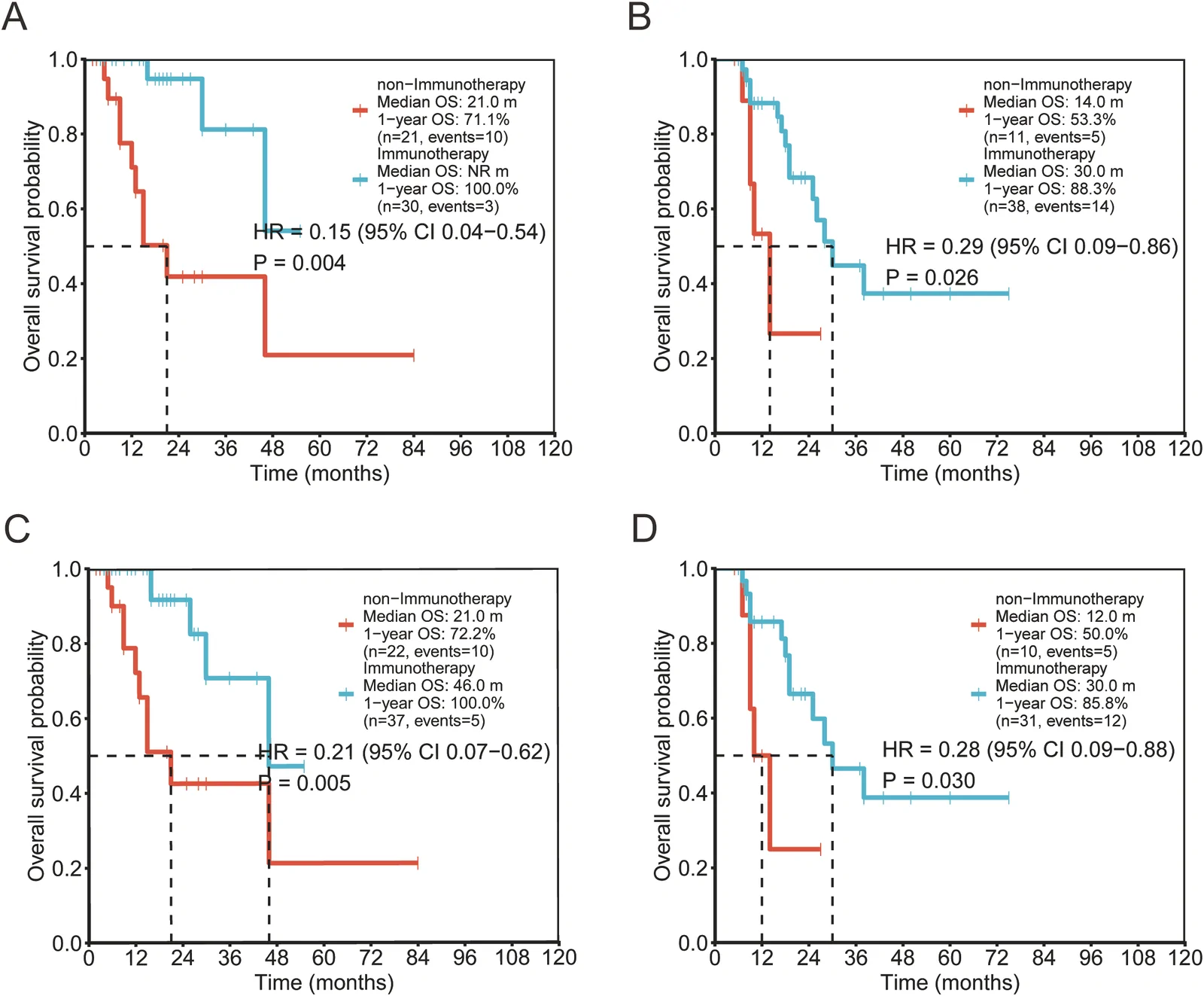
ICI therapy improved overall survival in non-surgically treated patients with SCLC across different disease stages. Kaplan–Meier analyses of overall survival (OS) in patients without surgical resection treated with or without immune checkpoint inhibitor (ICI) therapy. Significant OS benefits with ICI treatment were observed in patients with LS-SCLC **(A)**, ES-SCLC **(B)**, stage III disease **(C)**, and stage IV disease **(D)**. In surgically treated patients, ICI therapy showed a trend toward prolonged OS in the LS and stage III subgroups, although the differences were not statistically significant. HR, hazard ratio; NR, not reached; OS, overall survival; ICI, immune checkpoint inhibitor.

Stratification by prior radiotherapy status demonstrated that, among patients without previous radiotherapy, ICI treatment significantly prolonged OS in both LS-SCLC (HR = 0.18, 95% CI: 0.06–0.62, P = 0.006; 1-year OS: 100.0% vs. 66.4%, **Figure 4A**) and ES-SCLC patients (30 months for ICI vs. 12 months for non-ICI, HR = 0.24, 95% CI: 0.07–0.89, P = 0.033; 1-year OS: 86.7% vs. 41.7%, **Figure 4B**). Similar survival benefits were consistently observed in stage III (46 months for ICI vs. 21 months for non-ICI, HR = 0.24, 95% CI: 0.08–0.69, P = 0.008; 1-year OS: 100% vs. 67.9%, **Figure 4C**) and stage IV patients (30 months for ICI vs. 10.5 months for non-ICI, HR = 0.10, 95% CI: 0.02–0.53, P = 0.007; 1-year OS: 84.6% vs. 25%, **Figure 4D**). Conversely, although prolonged mOS was observed in LS, ES, and stage III–IV patients with prior radiotherapy exposure, none of these differences reached statistical significance. Because some patients received radiotherapy after disease progression, radiotherapy exposure differed between the PFS and OS analyses. **Table 1** reports radiotherapy status for the PFS analysis. For the OS analysis, radiotherapy was administered to 32 of 76 patients in the ICI group and 21 of 66 patients in the non-ICI group.

**Figure 4:**
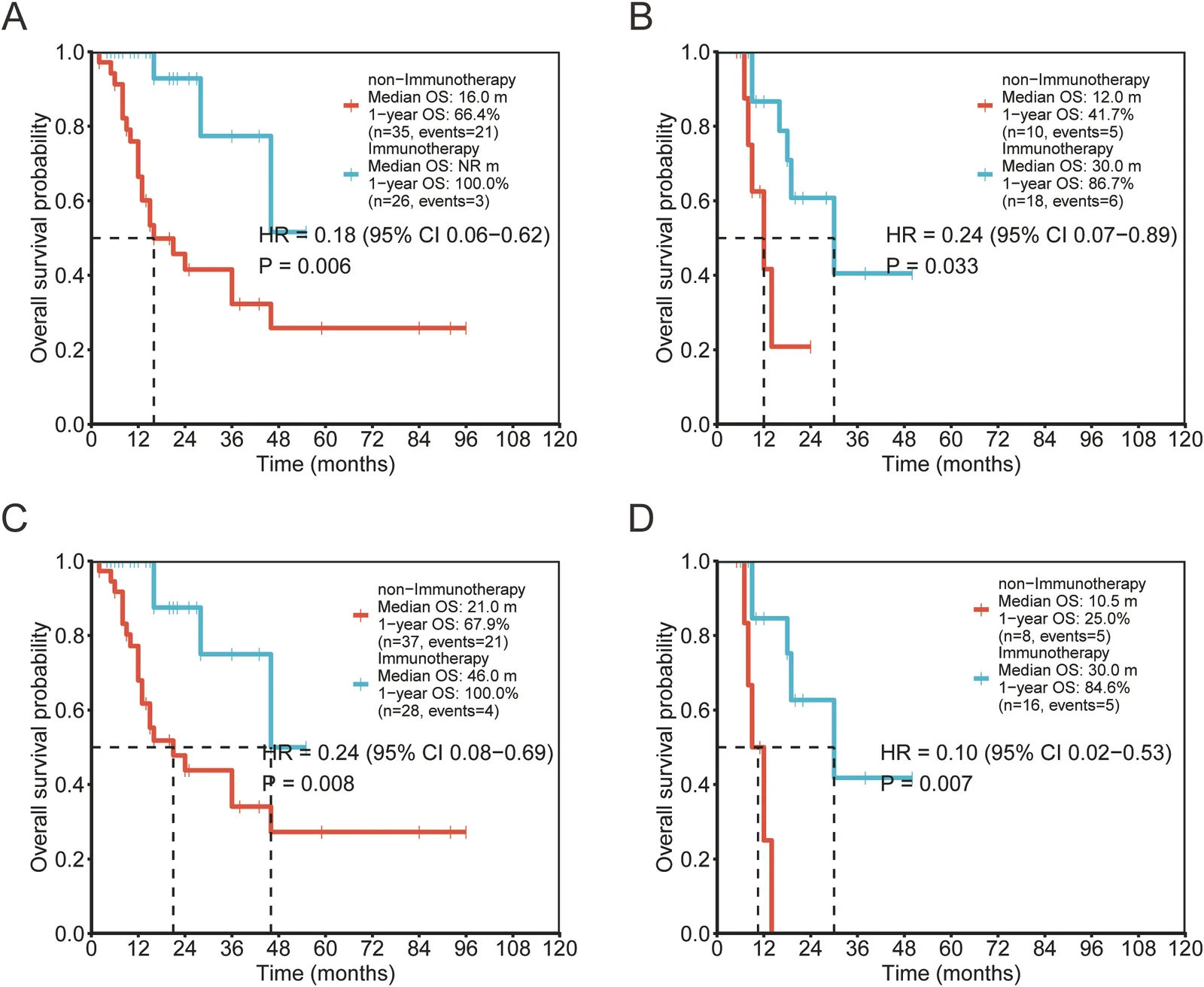
ICI therapy improved overall survival in patients with SCLC without prior radiotherapy across different disease stages. Kaplan–Meier analyses of overall survival (OS) in patients without previous radiotherapy treated with or without immune checkpoint inhibitor (ICI) therapy. Significant OS benefits with ICI treatment were observed in patients with LS-SCLC **(A)**, ES-SCLC **(B)**, stage III disease **(C)**, and stage IV disease **(D)**. Although prolonged OS was also observed in patients with prior radiotherapy exposure, the differences were not statistically significant. HR, hazard ratio; NR, not reached; OS, overall survival; ICI, immune checkpoint inhibitor.

In the multivariable Cox proportional hazards regression analysis for OS, age, sex, T stage, N stage, M stage, surgery, radiotherapy, and immunotherapy were included as covariates. After adjustment for these factors, immunotherapy remained independently associated with improved OS (HR = 0.27, 95% CI: 0.12–0.57, P < 0.001). In contrast, M stage was independently associated with an increased risk of death (HR = 3.12, 95% CI: 1.56–6.22, P = 0.001). Age, sex, T stage, N stage, surgery, and radiotherapy were not significantly associated with OS in the multivariable model (**Table 2**).

**Table 2:** Multivariable Cox regression analysis for overall survival.

Variable	HR	95% CI	P value
age	1.00	0.97–1.04	0.905
Sex	1.13	0.60–2.13	0.695
T-stage	1.08	0.83–1.41	0.553
N-stage	1.30	0.86–1.97	0.206
M-stage	3.12	1.56–6.22	0.001
Surgery	0.95	0.45–1.97	0.883
Radiation	0.63	0.34–1.17	0.145
Immunotherapy	0.27	0.12–0.57	<0.001

### ICI treatment improved PFS across clinical subgroups

ICI treatment demonstrated an overall trend toward improved PFS across different disease stages and treatment subgroups, with statistically significant benefits primarily observed in patients with stage IV and ES disease. Based on VALSG staging, patients with LS disease exhibited mPFS values of 18 months for ICI vs 11 months for non-ICI (HR = 0.76, 95% CI: 0.43–1.33, P = 0.335, **Figure 5A**), with corresponding 1-year PFS rates of 55.0% and 47.2%, respectively. In patients with ES disease, mPFS was 10 months for ICI vs 5 months for non-ICI (HR = 0.36, 95% CI: 0.19–0.71, P = 0.003, **Figure 5B**), with corresponding 1-year PFS rates of 31.5% and 11.8%, respectively. According to TNM staging, mPFS in stage III patients was 16 months for ICI vs 11 months for non-ICI (HR = 0.74, 95% CI: 0.43–1.26, P = 0.265, **Figure 5C**), with corresponding 1-year PFS rates of 52.7% and 45.7%, respectively. In stage IV patients, mPFS was 9 months for ICI vs 5 months for non-ICI (HR = 0.31, 95% CI: 0.15–0.65, P = 0.002, **Figure 5D**), with corresponding 1-year PFS rates of 31.2% and 14.3%, respectively.

**Figure 5:**
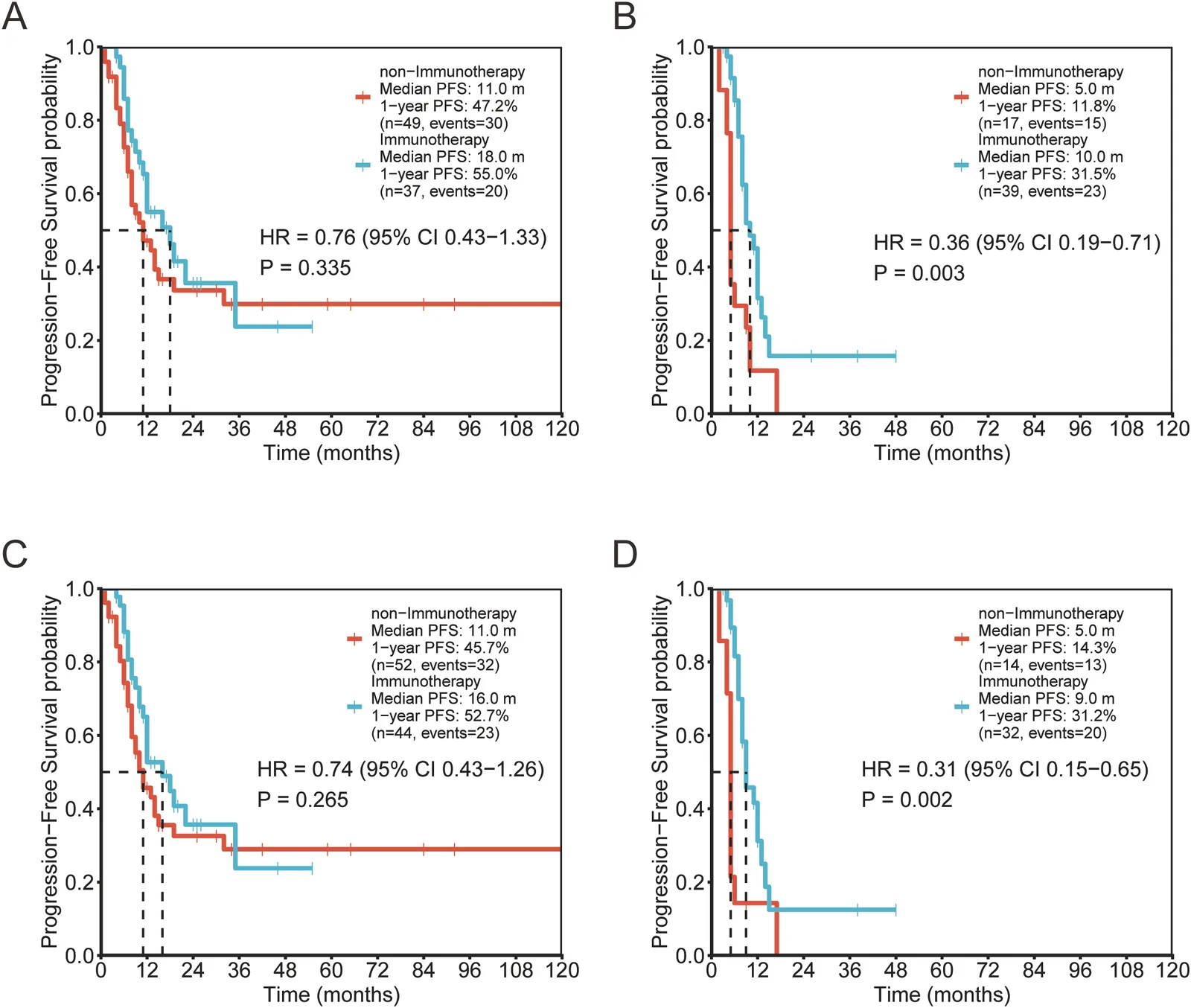
ICI therapy improved progression-free survival primarily in patients with ES-SCLC and stage IV disease. Kaplan–Meier analyses of progression-free survival (PFS) in patients treated with or without immune checkpoint inhibitor (ICI) therapy. Significant PFS benefits with ICI treatment were observed in patients with ES-SCLC **(B)** and stage IV disease **(D)**, while patients with LS-SCLC **(A)** and stage III disease **(C)** showed a trend toward prolonged PFS that did not reach statistical significance. HR, hazard ratio; PFS, progression-free survival; ICI, immune checkpoint inhibitor.

Further subgroup analyses demonstrated that, among patients who did not receive radiotherapy, ICI treatment consistently improved PFS, with statistically significant benefits primarily observed in patients with stage IV or ES disease. In stage III patients, mPFS was 12 months for ICI vs 9 months for non-ICI (HR = 0.72, 95% CI: 0.40–1.29, P = 0.271, **Figure 6A**), with corresponding 1-year PFS rates of 47.6% and 36.6%, respectively. In patients with LS disease, mPFS was 12 months for ICI vs 8 months for non-ICI (HR = 0.72, 95% CI: 0.39–1.31, P = 0.283, **Figure 6B**), with corresponding 1-year PFS rates of 49.1% and 36.7%, respectively. Due to the relatively shorter survival duration in advanced-stage disease, 6-month PFS rates were additionally evaluated in stage IV and ES subgroups. Stage IV patients exhibited mPFS values of 9 months for ICI vs 5 months for non-ICI (HR = 0.18, 95% CI: 0.06–0.51, P = 0.001, **Figure 6C**), with corresponding 6-month PFS rates of 88.9% and 12.5%, respectively, while patients with ES disease demonstrated mPFS values of 10 months for ICI vs 5 months for non-ICI (HR = 0.29, 95% CI: 0.11–0.75, P = 0.011, **Figure 6D**), with corresponding 6-month PFS rates of 90.5% and 30%, respectively. In contrast, although a trend toward improved PFS was also observed among patients who had previously received radiotherapy, none of these differences reached statistical significance.

**Figure 6:**
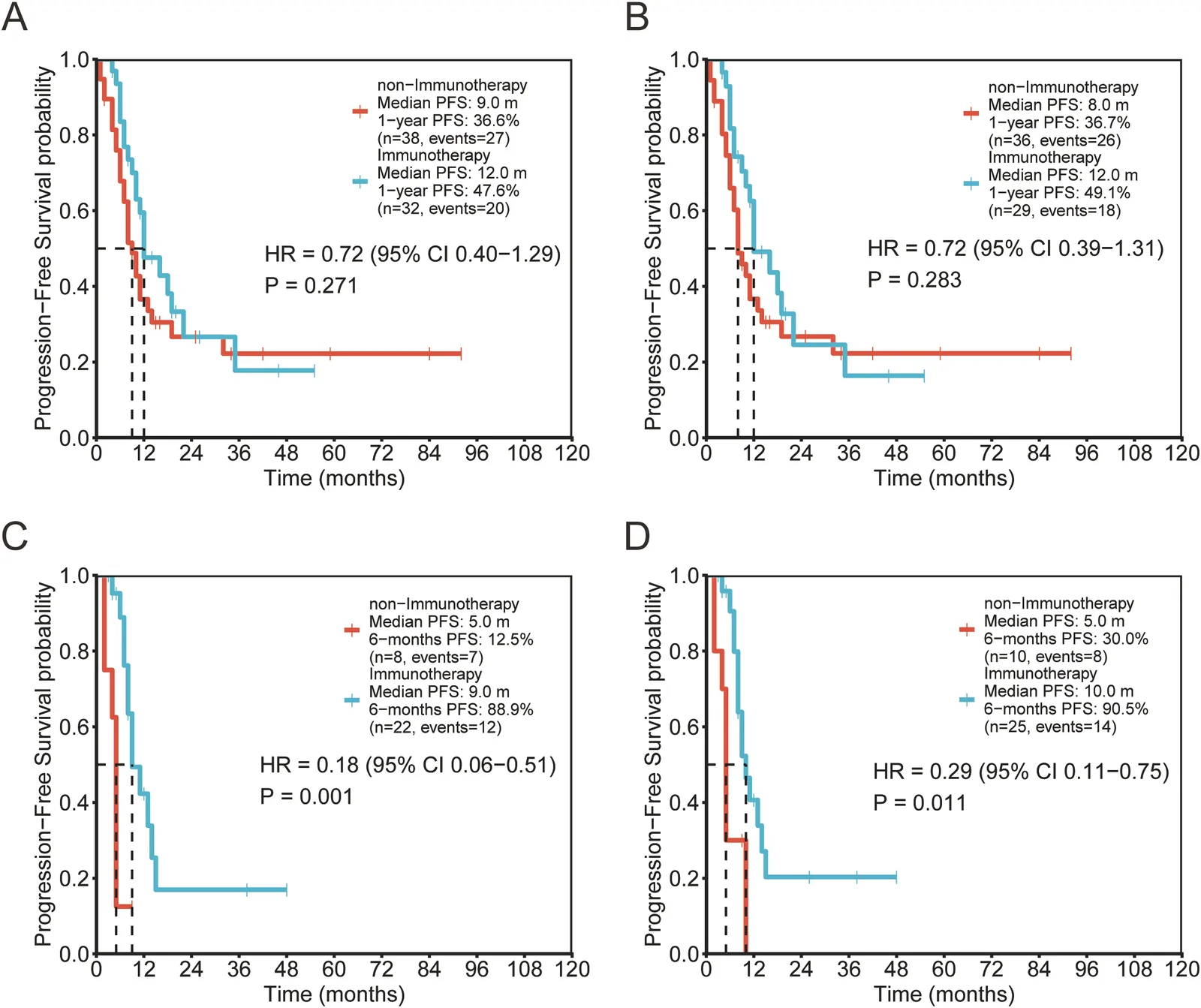
ICI therapy improved progression-free survival in patients with SCLC without prior radiotherapy, particularly in ES-SCLC and stage IV disease. Kaplan–Meier analyses of progression-free survival (PFS) in patients without previous radiotherapy treated with or without immune checkpoint inhibitor (ICI) therapy. Significant PFS benefits with ICI treatment were observed in stage IV **(C)** and ES-SCLC **(D)** patients, while patients with stage III disease **(A)** and LS-SCLC **(B)** showed a trend toward prolonged PFS without statistical significance. Although similar trends were observed in patients with prior radiotherapy exposure, the differences were not statistically significant. HR, hazard ratio; PFS, progression-free survival; ICI, immune checkpoint inhibitor.

Subgroup analysis based on surgical status also suggested a potential benefit of ICI therapy. In patients who did not undergo surgery, ICI treatment also showed a trend toward improved PFS in the stage III, stage IV, LS and ES subgroups. Specifically, stage III patients demonstrated mPFS values of 12 months for ICI vs 8 months for non-ICI (HR = 0.63, 95% CI: 0.31–1.26, P = 0.19, **Figure 7A**), with corresponding 1-year PFS rates of 45.1% and 48.1%, respectively. LS patients exhibited mPFS values of 12 months for ICI vs 8 months for non-ICI (HR = 0.61, 95% CI: 0.30–1.27, P = 0.187, **Figure 7B**), with corresponding 1-year PFS rates of 46.5% and 45.1%, respectively. Stage IV patients exhibited mPFS values of 9 months for ICI vs 5 months for non-ICI (HR = 0.28, 95% CI: 0.13–0.63, P = 0.002, **Figure 7C**), with corresponding 1-year PFS rates of 32.5% and 10%, respectively, while ES patients demonstrated mPFS values of 11 months for ICI vs 5 months for non-ICI (HR = 0.32, 95% CI: 0.15–0.69, P = 0.004, **Figure 7D**), with corresponding 1-year PFS rates of 32.6% and 18.2%, respectively. Overall, ICI treatment demonstrated a consistent trend toward prolonged PFS across multiple clinical subgroups, with statistically significant benefits primarily observed in stage IV and ES disease. In contrast, although a trend toward improved PFS was also observed among patients who had previously undergone surgical resection, none of these differences reached statistical significance.

**Figure 7:**
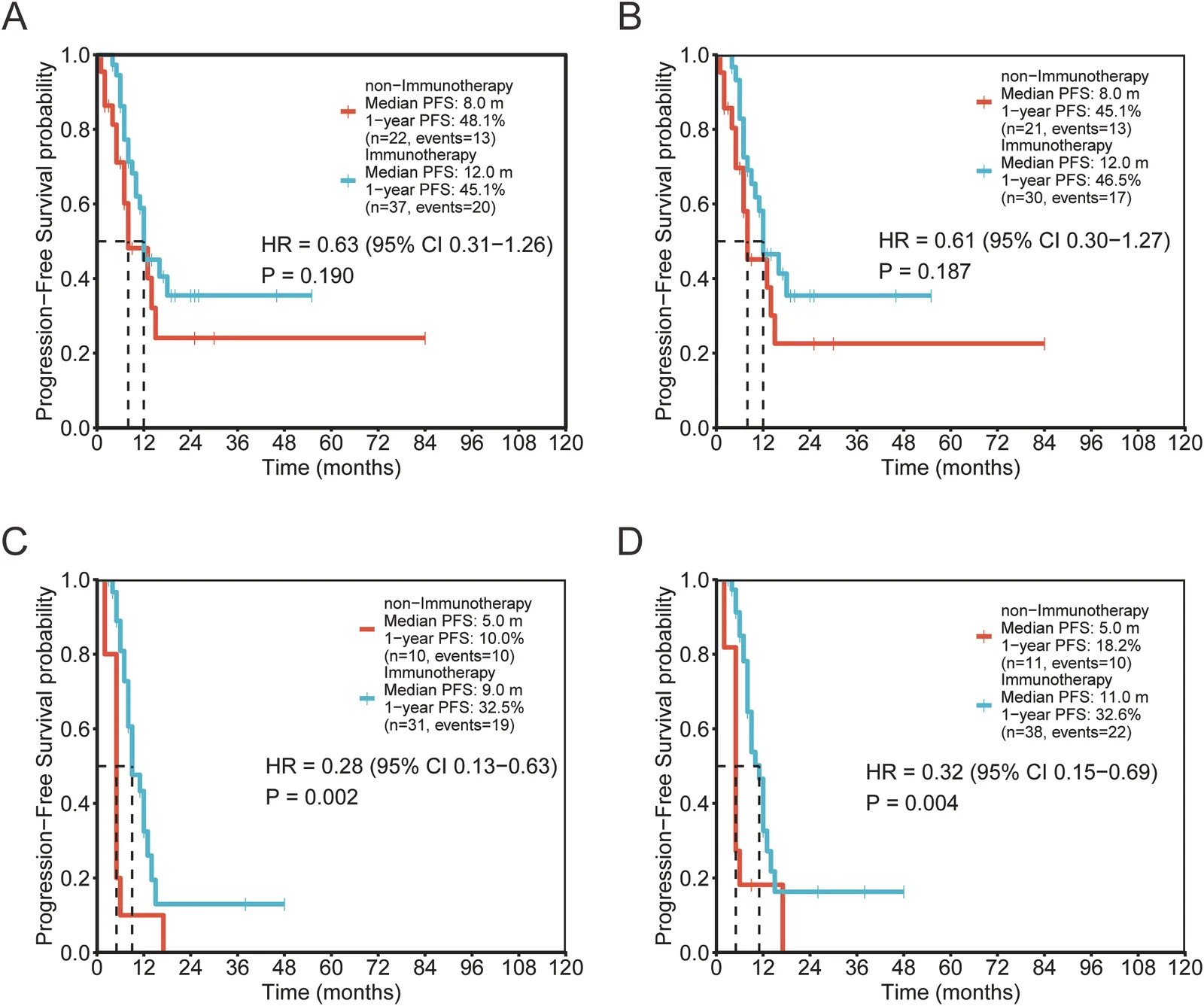
Progression-free survival according to disease stage in patients with SCLC who did not undergo surgical resection. Kaplan–Meier analyses of progression-free survival (PFS) comparing patients treated with immune checkpoint inhibitor (ICI)-based therapy and those receiving non-ICI therapy. **(A)** Stage III disease; **(B)** limited-stage (LS) SCLC; **(C)** stage IV disease; and **(D)** extensive-stage (ES) SCLC. ICI therapy was associated with significantly prolonged PFS in patients with stage IV and ES disease, whereas no statistically significant differences were observed in the stage III or LS subgroups. HR, hazard ratio; CI, confidence interval; PFS, progression-free survival; ICI, immune checkpoint inhibitor; LS, limited stage; ES, extensive stage.

In the multivariable Cox proportional hazards regression analysis for PFS, immunotherapy was independently associated with a lower risk of disease progression or death after adjustment for age, sex, T stage, N stage, M stage, surgery, and radiotherapy (HR = 0.36, 95% CI: 0.21–0.63, P < 0.001). M stage was independently associated with poorer PFS (HR = 2.49, 95% CI: 1.50–4.13, P < 0.001), whereas radiotherapy was associated with a reduced risk of progression or death (HR = 0.57, 95% CI: 0.34–0.96, P = 0.034). Age, sex, T stage, N stage, and surgery were not statistically significant in the adjusted model (**Table 3**).

**Table 3:** Multivariable Cox regression analysis for progression-free survival.

Variable	HR	95% CI	P value
age	1.01	0.98–1.04	0.635
Sex	1.12	0.69–1.83	0.637
T-stage	1.03	0.83–1.28	0.771
N-stage	1.13	0.81–1.58	0.461
M-stage	2.49	1.50–4.13	<0.001
Surgery	1.70	0.95–3.03	0.074
Radiation	0.57	0.34–0.96	0.034
Immunotherapy	0.36	0.21–0.63	<0.001

### Association of ICI therapy with 12-month ORR across clinical subgroups

At the prespecified 12-month response assessment, ICI treatment was associated with numerically higher ORRs across all disease-stage subgroups. Among patients with stage III disease and LS-SCLC, the ORRs were higher in the ICI group than in the non-ICI group (59.1% vs. 50.0%, P = 0.373; and 59.5% vs. 51.0%, P = 0.436, respectively), although these differences were not statistically significant. Similarly, a higher ORR was observed in patients with stage IV disease (43.8% vs. 14.3%, P = 0.092), but the difference did not reach statistical significance. In patients with ES-SCLC, ICI treatment was associated with a significantly higher ORR than non-ICI treatment (46.2% vs. 17.6%, P = 0.043) (**Table 4**).

**Table 4:** Association of immunotherapy with 12-month ORR according to disease stage in SCLC.

Group	n	Responders/total	ORR, % (95%CI)	Statistical analysis	χ²	*p*
III stage						
ICI	44	26/44	59.1(43.2-73.7)	Pearsonχ²	0.793	0.373
Non- ICI	52	26/52	50.0(35.8-64.2)			
Limited Stage						
ICI	37	22/37	59.5(42.1-75.2)	Pearsonχ²	0.606	0.436
Non- ICI	49	25/49	51.0(36.3-65.6)			
IV stage						
ICI	32	14/32	43.8(26.4-62.3)	Fisher exact	–	0.092
Non- ICI	14	2/14	14.3(1.8-42.8)			
Extensive Stage						
ICI	39	18/39	46.2(30.1-62.8)	Pearsonχ²	4.105	0.043
Non- ICI	17	3/17	17.6(3.8-43.4)			

ORRs are presented as proportions with 95% confidence intervals (CIs), calculated using the exact Clopper–Pearson method. Between-group comparisons were performed using Pearson’s chi-square test without continuity correction or Fisher’s exact test, as appropriate. All tests were two-sided, and P < 0.05 was considered statistically significant.

Subgroup analyses according to surgical status showed that, among patients who underwent surgery, ICI treatment was associated with significantly higher ORRs in both the stage III subgroup (100% vs. 46.7%, P = 0.012) and the LS subgroup (100% vs. 50.0%, P = 0.027). Among patients who did not undergo surgical resection, higher ORRs were observed with ICI treatment in the stage IV (45.2% vs. 10.0%, P = 0.063) and ES (47.4% vs. 18.2%, P = 0.162) subgroups, although neither difference reached statistical significance. No significant between-group differences were observed in the stage III or LS subgroups (**Table 5**).

**Table 5:** 12-month ORR to immunotherapy stratified by surgical resection status and disease stage in SCLC.

Category	Group	n	Responders/total	ORR, % (95%CI)	Statistical analysis	χ²	*p*
Surgery							
Stage III	ICI	7	7/7	100 (59.0-100.0)	Fisher exact	–	0.012
	Non- ICI	30	14/30	46.7 (28.3-65.7)			
Limited Stage	ICI	7	7/7	100 (59.0-100.0)	Fisher exact	–	0.027
	Non- ICI	28	14/28	50.0 (30.6-69.4)			
Non-surgery							
Stage IV	ICI	31	14/31	45.2 (27.3-64.0)	Fisher exact	–	0.063
	Non- ICI	10	1/10	10.0 (0.3-44.5)			
Extensive Stage	ICI	38	18/38	47.4 (31.0-64.2)	Fisher exact	–	0.162
	Non- ICI	11	2/11	18.2 (2.3-51.8)			
Stage III	ICI	37	19/37	51.4 (34.4-68.1)	Pearsonχ²	0.056	0.812
	Non- ICI	22	12/22	54.5 (32.2-75.6)			
Limited Stage	ICI	30	15/30	50.0 (31.3-68.7)	Pearsonχ²	0.028	0.867
	Non- ICI	21	11/21	52.4 (29.8-74.3)			

ORRs are presented as proportions with 95% confidence intervals (CIs), calculated using the exact Clopper–Pearson method. Between-group comparisons were performed using Pearson’s chi-square test without continuity correction or Fisher’s exact test, as appropriate. All tests were two-sided, and P < 0.05 was considered statistically significant.

Further stratification by radiotherapy status showed that, among patients who did not receive radiotherapy, numerically higher ORRs were observed with ICI treatment in the stage IV (54.5% vs. 12.5%, P = 0.092) and ES (52.0% vs. 20.0%, P = 0.134) subgroups, although neither difference was statistically significant. Numerical differences were also observed in the stage III and LS subgroups without statistical significance. Among patients who received radiotherapy, no statistically significant between-group differences in 12-month ORR were observed across any of the analyzed disease-stage subgroups (**Table 6**).

**Table 6:** 12-month ORR to immunotherapy stratified by radiotherapy status and disease stage in SCLC.

Category	Group	n	Responders/total	ORR, % (95%CI)	Statistical analysis	χ²	*p*
Non-radiotherapy							
Stage IV	ICI	22	12/22	54.5 (32.2-75.6)	Fisher exact	–	0.092
	Non- ICI	8	1/8	12.5 (0.3-52.7)			
Extensive Stage	ICI	25	13/25	52.0 (31.3-72.2)	Fisher exact	–	0.134
	Non- ICI	10	2/10	20.0 (2.5-55.6)			
Stage III	ICI	32	17/32	53.1 (34.7-70.9)	Pearsonχ²	1.305	0.253
	Non- ICI	38	15/38	39.5 (24.0-56.6)			
Limited Stage	ICI	29	16/29	55.2 (35.7-73.6)	Pearsonχ²	1.714	0.191
	Non- ICI	36	14/36	38.9 (23.1-56.5)			
Radiotherapy							
Stage IV	ICI	10	2/10	20.0 (2.5-55.6)	Fisher exact	–	1.000
	Non- ICI	6	1/6	16.7 (0.4-64.1)			
Extensive Stage	ICI	14	5/14	35.7 (12.8-64.9)	Fisher exact	–	0.613
	Non- ICI	7	1/7	14.3 (0.4-57.9)			
Stage III	ICI	12	9/12	75.0 (42.8-94.5)	Fisher exact	–	1.000
	Non- ICI	14	11/14	78.6 (49.2-95.3)			
Limited Stage	ICI	8	6/8	75 (34.9-96.8)	Fisher exact	–	0.618
	Non- ICI	13	11/13	84.6 (54.6-98.1)			

ORRs are presented as proportions with 95% confidence intervals (CIs), calculated using the exact Clopper–Pearson method. Between-group comparisons were performed using Pearson’s chi-square test without continuity correction or Fisher’s exact test, as appropriate. All tests were two-sided, and P < 0.05 was considered statistically significant.

## Discussion

In this retrospective study, we systematically evaluated the clinical efficacy of ICI based therapy in patients with SCLC across different disease stages and treatment backgrounds. Our findings demonstrated that ICI therapy was associated with improved OS, PFS, and ORR in multiple clinical subgroups, with the most consistent and statistically significant benefits observed in patients with stage IV and ES disease.

Previous landmark phase III trials, including IMpower133 and CASPIAN, established PD-L1 inhibitor–based immunotherapy combined with platinum-etoposide chemotherapy as the standard first-line treatment for extensive-stage small-cell lung cancer (ES-SCLC) (8, 13). More recently, the phase III ADRIATIC trial demonstrated that consolidation durvalumab after concurrent chemoradiotherapy significantly improved both OS and PFS in patients with limited-stage SCLC (LS-SCLC), providing prospective evidence for the clinical benefit of immunotherapy in this population (14). However, real-world evidence regarding the efficacy of ICIs in patients with LS disease remains limited. In the present study, survival improvements associated with ICI therapy were observed in both LS- and ES-SCLC, suggesting that the clinical benefits of immunotherapy may extend beyond traditionally recognized advanced-stage populations.

Notably, although favorable survival trends were observed across different disease stages, statistically significant benefits were more consistently identified in patients with stage IV and extensive-stage disease. The biological basis for this observation remains uncertain. Previous studies have suggested that advanced tumors may exhibit increased tumor mutational burden and neoantigen load, which could potentially enhance tumor immunogenicity and responsiveness to immune checkpoint blockade (7, 15, 16). However, TMB and neoantigen burden were not assessed in our cohort; therefore, this explanation should be regarded only as a hypothesis derived from previous literature rather than a mechanism demonstrated by the present study. In addition, the poorer baseline prognosis and higher event rates typically observed in advanced-stage disease may have increased the statistical detectability of treatment-associated survival differences. Accordingly, the apparently more pronounced benefit observed in advanced-stage subgroups should be interpreted cautiously and requires confirmation in larger prospective studies with integrated biomarker analyses.

Subgroup analyses according to surgical and radiotherapy status further highlighted the context-dependent efficacy of immune checkpoint inhibitor (ICI) treatment. Among patients who did not undergo surgery or radiotherapy, ICI therapy showed more pronounced benefits in OS, PFS, and ORR, particularly in advanced-stage disease. These findings may, in part, be related to differences in tumor burden and the baseline immune microenvironment (7). These findings suggest that immunotherapy may be associated with additional clinical benefit in selected patients who did not receive definitive local treatment. In contrast, among patients who had previously undergone surgery or radiotherapy, survival improvements associated with ICI therapy did not consistently reach statistical significance, which may be attributable to limited statistical power, treatment selection bias, and confounding effects related to prior local disease control (8, 11). It should also be noted that radiotherapy was not uniformly administered at baseline, and some patients received radiotherapy after disease progression. Therefore, radiotherapy status may partly reflect post-baseline treatment exposure rather than a fixed pretreatment characteristic, which may have introduced additional time-dependent and treatment-selection bias into the radiotherapy-stratified analyses.

Interestingly, despite the lack of statistical significance in some analyses, surgically treated patients still demonstrated prolonged survival and favorable PFS trends following ICI therapy, indicating a potential role for immunotherapy in perioperative or multimodal treatment strategies for selected LS-SCLC patients (7). In addition, higher ORRs with ICI therapy were mainly observed in the stage IV and ES subgroups, broadly consistent with the survival analyses. These findings suggest a possible greater benefit in advanced-stage SCLC, although they should be interpreted cautiously given the subgroup sample sizes and potential confounding (8).

Several limitations should be acknowledged. First, the retrospective single-center design and long enrollment period may have introduced selection bias, limited generalizability, and treatment-era bias. Second, baseline imbalances, particularly in disease stage and surgical status, remained despite multivariable adjustment, and residual confounding cannot be excluded. Performance status, comorbidity data, and detailed radiotherapy characteristics were also incompletely available.

Third, requiring at least three cycles of first-line ICI therapy for group assignment may have introduced guaranteed-survival or immortal-time bias. In addition, radiotherapy timing was heterogeneous, with some patients receiving radiotherapy after progression, potentially introducing further time-dependent and treatment-selection bias.

Finally, several subgroup analyses included small and overlapping populations, and treatment regimens and biomarker data were heterogeneous or incomplete. Therefore, these subgroup findings should be regarded as exploratory and hypothesis-generating.

Despite these limitations, this study provides real-world evidence of an association between ICI-based therapy and improved clinical outcomes in SCLC. The findings require validation in larger prospective multicenter studies.

## Conclusions

ICI-based therapy was associated with improved OS, PFS, and 12-month ORR in several clinical subgroups of SCLC, with more consistent favorable associations observed in stage IV and extensive-stage disease. Exploratory analyses according to surgical and radiotherapy status also suggested differences in treatment-associated outcomes; however, these findings should be interpreted cautiously because of small subgroup sizes, treatment-selection bias, and residual confounding. Larger prospective multicenter studies are warranted to validate these observations.

## Data Availability

The original contributions presented in the study are included in the article/supplementary material. Further inquiries can be directed to the corresponding authors.
